# Case of Aneurism of Orbit

**Published:** 1887-12

**Authors:** A. W. Prichard

**Affiliations:** Surgeon to the Bristol Royal Infirmary


### CASE OF ANEURISM OF ORBIT. By A. W. Prichard, M.R.C.S., Surgeon to the Bristol Royal Infirmary.

I wish to record very briefly a case of traumatic aneurism of the orbit, treated eventually by ligation of the common carotid, in which the treatment has been partly, but not wholly, successful.

James Sparks, æt. 10, was looking through a keyhole on the 23rd of May, when a boy at the other side of the door pushed a piece of umbrella-wire through the keyhole into his eye. The wire, probably, was thrust deep into the orbit by the inner side of the eyeball. He was taken to the Infirmary at once, and detained. A small wound on the conjunctiva, and some blood under it towards the inner side, were the most noticeable signs of the injury. A few hours afterwards there was considerable ecchymosis; the globe was almost fixed, moving from side to side only about $\frac{1}{8}$ of an inch; this, of course, was the cause of double vision, and, excepting that, the sight of the injured eye was unaffected.

During the first fortnight the ecchymosis was the only symptom—both lids were very dark, and the ocular conjunctiva had blood under it. The boy complained very little, and a cold pad was kept generally over the eye. By June 9th, however, I noticed that there was some protrusion, and a faint pulsation could be felt on putting the hand over the eyeball. On listening with a stethoscope on the eye, a loud bruit could be heard; this was audible all over that side, and faintly over the other side of the cranium. Moreover the boy complained of a blowing sound in the left ear.

Thinking the case was one of traumatic aneurism—or possibly of a blood-tumour pressing on the carotid artery, for three weeks I tried the effect of continuous pressure on the eyeball and ice around the orbit. This had the effect of ameliorating the diplopia—but accurate measurement from the forehead proved that the protrusion of the globe was increasing.

By July 4th the protrusion had increased so that the injured eye was as much as $\frac{11}{16}$ of an inch in front of the other. There was considerable chemosis, and blueness all round the orbit; the bruit was very marked, and pressure on the left carotid entirely stopped it; and the boy had headaches and restless nights. Accordingly, after consultation, on July 5th. I ligatured the common carotid on that side at the level of the cricoid cartilage. I had no great difficulty in the operation—the vein was large, and as it were enveloping the artery, and it had to be held carefully out of the way. I tied the vessel with medium-sized catgut, and put a small drainage tube in the wound, but did the operation without a spray.

In the evening after the operation no bruit could be heard and no pulsation felt, and the eyeball had receded

$\frac{1}{8}$ of an inch. During the next week the boy went on very well. The wound healed kindly and the eye receded, the double vision was less, and the chemosis and swelling of tissues round the orbit went down.

I kept him in the Infirmary for six weeks after, and was much disappointed at the return of the bruit, which was heard as early as one week after the operation—however, it was not nearly so loud, and the boy could not hear it himself. The eyeball receded as gradually as it had come forward, and when the patient went out the protrusion only amounted to $\frac{3}{16}$ of an inch: the diplopia was still present—otherwise the boy was well.

He is now much in the same condition as then—his eye is red and prominent—*there is still a bruit*, but no pulsation.

